# Strategy drives implementation: COVID vaccination in Israel

**DOI:** 10.1186/s13584-021-00445-1

**Published:** 2021-02-01

**Authors:** Sherry Glied

**Affiliations:** Robert F. Wagner Graduate School of Public Service, New York University, New York, USA

**Keywords:** COVID-19, Comparative health systems, United States government

## Abstract

This commentary on Rosen, Waitzberg, and Israeli’s assessment of Israel’s COVID response points to differences in the coherence of each national government’s strategy as the key factor influencing the effectiveness of the vaccine drive. The strengths of the Israeli healthcare system facilitated implementation of the roll-out, but the government’s unambiguous prioritization of vaccination drove implementation success.

## Main text

In ordinary times, the Israeli healthcare system, with its low costs, excellent outcomes, and high levels of consumer satisfaction functions quite effectively. In its rollout of COVID-19 vaccination, it is unambiguously the global leader.

Bruce Rosen, Ruth Waitzberg, and Avi Israeli have laid out a broad set of features of the Israeli rollout that have led to its success [1]. These include Israel’s centralized structure (which eliminated the need to coordinate with sub-national governments) and its small size (which made procuring vaccine for the entire population more feasible). I want to focus on one broad key difference between the Israeli and US experiences that has shaped the differences in trajectories. The Israeli government, however fractionated on other issues, was unified in approaching COVID vaccination as a crucial, urgent response to a nation-threatening emergency. The US Federal government had no such unified vision. It is certainly true that the Israeli healthcare system was well organized to support the government’s strategy. The disappointing experience to date in the US, however, has more to do with the lack of such an overarching strategy than with the any of the many weaknesses of the healthcare system itself.

Israel’s view of COVID vaccination as mission critical gave the government latitude to make large-scale vaccine procurement deals with Pfizer (and later Moderna) without worrying too much about the pricing or data-sharing arrangements in the deal. The government sensibly recognized that the ongoing costs, both economic and social, of the continuing pandemic were so great that any vaccine overpayment would quickly be recouped through economic growth – and would surely be forgiven by the public. The ongoing involvement of internal security agencies in Israeli contact tracing efforts (unthinkable in the US) likely muted any concerns about any sharing of aggregated data with drug companies.

The sense of urgency extended into the rollout. While authorities in the US agonized over the ethical implications of the vaccine prioritization list, settling on a set of parameters far too complicated for implementation and then nearly immediately readjusting them, Israel proceeded with a straightforward, age-based criterion. Consistent with a sense of social solidarity around the effort, infractions of the priority rules (vaccination of adult children bringing elderly parents to vaccination sites, for example) were treated as understandable and forgivable. In the US, similar slips, mainly associated with the need to use up open vials of vaccine, generated disproportionate recriminations. The sanguine attitude in Israel was certainly helped by the sense that the timetable for complete national vaccination was short and that line-jumpers were not gaining much of an advantage.

The final piece of the strategy was the experience and salience for the Israeli healthcare system and society of responding to national emergencies. While pandemic preparation has been a staple of local government planning and JCAHO accreditation in the US, and was taken very seriously in the years immediately following 9/11, the exercise had become more perfunctory as emergencies seemed farther away. The Israeli systems never had the luxury of such complacency.

Israel’s ability to quickly roll out its COVID strategy is testimony to the strength and resilience of its healthcare system. But it’s difficult to draw broader lessons from it, as the situation is unprecedented and, we hope, unlikely to be repeated. The more general lesson, however, is the importance of a unified strategy in recognizing and responding to a national emergency. Let’s hope that the incoming Biden administration sees Israel’s experience as an exemplar of that lesson.

## Data Availability

Not applicable.
